# Glucagon-like peptide 1 receptor agonist use and risk of thyroid cancer: Scandinavian cohort study

**DOI:** 10.1136/bmj-2023-078225

**Published:** 2024-04-10

**Authors:** Björn Pasternak, Viktor Wintzell, Anders Hviid, Björn Eliasson, Soffia Gudbjörnsdottir, Christian Jonasson, Kristian Hveem, Henrik Svanström, Mads Melbye, Peter Ueda

**Affiliations:** 1 Clinical Epidemiology Division, Department of Medicine, Solna, Karolinska Institutet, Stockholm, Sweden; 2 Department of Epidemiology Research, Statens Serum Institut, Copenhagen, Denmark; 3 Pharmacovigilance Research Center, Department of Drug Design and Pharmacology, Faculty of Health and Medical Sciences, University of Copenhagen, Copenhagen, Denmark; 4 Department of Medicine, Sahlgrenska University Hospital, Gothenburg, Sweden; 5 The Swedish National Diabetes Register, Västra Götalandsregionen, Gothenburg, Sweden; 6 Department of Molecular and Clinical Medicine, Institute of Medicine, University of Gothenburg, Gothenburg, Sweden; 7 HUNT Center for Molecular and Clinical Epidemiology, Department of Public Health and Nursing, Faculty of Medicine and Health Science, NTNU—Norwegian University of Science and Technology, Trondheim, Norway; 8 HUNT Research Center, Faculty of Medicine, NTNU—Norwegian University of Science and Technology, Levanger, Norway; 9 Department of Clinical Medicine, University of Copenhagen, Copenhagen, Denmark; 10 Department of Genetics, Stanford University School of Medicine, Stanford, CA, USA; 11 Danish Cancer Institute, Copenhagen, Denmark

## Abstract

**Objective:**

To investigate whether use of glucagon-like peptide 1 (GLP1) receptor agonists is associated with increased risk of thyroid cancer.

**Design:**

Scandinavian cohort study.

**Setting:**

Denmark, Norway, and Sweden, 2007-21.

**Participants:**

Patients who started GLP1 receptor agonist treatment were compared with patients who started dipeptidyl peptidase 4 (DPP4) inhibitor treatment, and in an additional analysis, patients who started sodium-glucose cotransporter 2 (SGLT2) inhibitor treatment.

**Main outcome measures:**

Thyroid cancer identified from nationwide cancer registers. An active-comparator new user study design was used to minimise risks of confounding and time related biases from using real world studies of drug effects. Cox regression was used to estimate hazard ratios, controlling for potential confounders with propensity score weighting.

**Results:**

The mean follow-up time was 3.9 years (standard deviation 3.5 years) in the GLP1 receptor agonist group and 5.4 years (standard deviation 3.5 years) in the DPP4 inhibitor group. 76 of 145 410 patients (incidence rate 1.33 events per 10 000 person years) treated with GLP1 receptor agonists and 184 of 291 667 patients (incidence rate 1.46 events per 10 000 person years) treated with DPP4 inhibitors developed thyroid cancer. GLP1 receptor agonist use was not associated with increased risk of thyroid cancer (hazard ratio 0.93, 95% confidence interval 0.66 to 1.31; rate difference −0.13, 95% confidence interval −0.61 to 0.36 events per 10 000 person years). The hazard ratio for medullary thyroid cancer was 1.19 (0.37 to 3.86). In the additional analysis comparing the GLP1 receptor agonist group with the SGLT2 inhibitor group, the hazard ratio for thyroid cancer was 1.16 (0.65 to 2.05).

**Conclusions:**

In this large cohort study using nationwide data from three countries, GLP1 receptor agonist use was not associated with a substantially increased risk of thyroid cancer over a mean follow-up of 3.9 years. In the main analysis comparing GLP1 receptor agonists with DPP4 inhibitors, the upper limit of the confidence interval was consistent with no more than a 31% increase in relative risk.

## Introduction

Concerns about thyroid cancer with glucagon-like peptide 1 (GLP1) receptor agonist use were first raised in the premarketing phase after studies showed increased rates of thyroid C cell tumours in rodents.^1^ While the relevance of these findings to humans is not known, in the United States, product labels of GLP1 receptor agonists include boxed warnings about thyroid cancer and these drugs are contraindicated in patients with a personal or family history of medullary thyroid cancer or multiple endocrine neoplasia type 2.

Subsequent reports based on pharmacovigilance data, post hoc analyses of randomised trials, and findings from observational studies indicate a potential link between GLP1 receptor agonists and thyroid cancer. In two analyses of spontaneous reports from the US Food and Drug Administration’s adverse event reporting system database, the reporting rates of thyroid cancer were 4.7 and 8 times higher, respectively, for GLP1 receptor agonists compared with other diabetes drugs.^2^ ^3^ In a meta-analysis based on 15 clinical trials that included at least one thyroid cancer outcome event, the odds ratio for thyroid cancer associated with GLP1 receptor agonists was 1.49 (95% confidence interval 0.83 to 2.66). In another meta-analysis based on 35 trials, which also included trials that had zero outcome events (of thyroid cancer), the risk ratio was 1.30 (95% confidence interval 0.86 to 1.97).^4^ ^5^ A nested case-control study that used US databases reported an odds ratio of 1.46 (95% confidence interval 0.98 to 2.19) for thyroid cancer when the GLP1 receptor agonist exenatide was compared with other diabetes drugs.^6^ More recently, a nested case-control study that used French health insurance data found a hazard ratio of 1.46 (95% confidence interval 1.23 to 1.74) for the association between thyroid cancer and current use of GLP1 receptor agonists compared with non-use of these drugs. Furthermore, the hazard ratio for medullary thyroid cancer was 1.78 (95% confidence interval 1.04 to 3.05) and the reporting ratio in a complementary pharmacovigilance analysis based on the World Health Organization’s database VigiBase was 30.5 (95% confidence interval 25.1 to 37.2) compared with other diabetes drugs.^7^ These data prompted the European Medicines Agency to raise a safety concern and start an investigation,^8^ which concluded that the available evidence does not support a causal association.^9^ We conducted a cohort study using nationwide data from three Scandinavian countries to investigate whether GLP1 receptor agonist use is associated with increased risk of thyroid cancer.

## Methods

We conducted a cohort study using healthcare and administrative registers in Denmark (2007-21), Norway (2010-18), and Sweden (2007-21), including population registers, prescription drug registers, national patient registers, and cancer registers, all with nationwide coverage in each country. These registers cover personal and vital status data, capture all prescriptions from all pharmacies nationwide, include diagnostic and procedure data from all outpatient specialist care and hospital admissions, and capture nearly all incident cancers.^10^

Patients aged 18-84 years were eligible for inclusion when they were new users of a GLP1 receptor agonist or a dipeptidyl peptidase 4 (DPP4) inhibitor; that is, patients who had not used either drug class at any time before cohort entry. Patients were excluded if they had thyroid cancer at any time before cohort entry, any other cancer in the previous year, end stage illness, human immunodeficiency virus infection, drug or alcohol misuse in the previous year, major pancreatic disease, genetic syndromes associated with thyroid cancer, and no healthcare contact in the previous year (to ensure a minimum level of activity in the healthcare system; healthcare contact defined as outpatient specialist care contact, hospital admission, or use of any prescription drug; definitions in etable 1).

The study design was an active-comparator new user design,^11^ with DPP4 inhibitors as the comparator. DPP4 inhibitors were introduced around the same time as the GLP1 receptor agonists, and have typically been used as second line agents. There are no known thyroid cancer signals with DPP4 inhibitors, although the hazard ratio from a recent French nested case-control study might be interpreted as a small increased risk with these drugs (1.10; 95% confidence interval 0.99 to 1.22).^7^ We conducted an additional analysis with sodium-glucose cotransporter 2 (SGLT2) inhibitors as the comparator for GLP1 receptor agonists; the study period for this analysis started in 2013 when SGLT2 inhibitors became available.

The outcome was thyroid cancer (international classification of diseases, 10th revision, code C73) identified from nationwide cancer registers. In additional analyses, we investigated thyroid cancer subtypes, including papillary, follicular, medullary, and other (etable 2).

### Statistical analysis

Patients were followed from start of drug use to the outcome event, emigration, death, or end of study period, whichever came first. Drug use was defined according to the observational analogue of intention to treat (ie, from the first filled prescription onwards).

We estimated hazard ratios using Cox regression, with days since start of treatment as the underlying time scale. Differences were considered statistically significant when the confidence intervals did not overlap 1.0. We examined the proportional hazards assumption by using a Wald test of the interaction between treatment status and time. A propensity score weighting method (fine stratification) was applied to control for confounding, which estimated the average treatment effect among treated patients. The propensity score was estimated using logistic regression as the probability of starting a GLP1 receptor agonist versus a DPP4 inhibitor conditional on a number of variables, including sociodemographic characteristics, medical history, other diabetes treatment, and markers of healthcare use, at cohort entry and stratified by country (etable 3). Patients with a propensity score outside the overlapping common distribution of the two groups and the highest 1% and lowest 1% of the common propensity score distribution were excluded (trimming); the propensity score was subsequently re-estimated and patients outside the common distribution of the new propensity score excluded. We then created 100 strata based on the propensity score distribution among patients treated with GLP1 receptor agonists; fine stratification weights were estimated according to Desai and colleagues.^12^ We calculated standardised differences to assess balance of baseline characteristics between groups, with a difference below 10% considered consistent with good balance. All reported rates, rate differences, cumulative incidences, and hazard ratios were adjusted using propensity score weighting.

We conducted several additional analyses. The main analysis estimated the hazard ratio for all available follow-up time, starting on day 1. In additional analyses, we estimated hazard ratios for the following time periods after the start of treatment: days 1-365, days ≥366, and days ≥731. The hazard ratio for the first year of follow-up was used to assess potential bias because an increased risk that is restricted to this period might not be biologically plausible. The hazard ratios for the periods covering days ≥366 and days ≥731 represented analyses with a lag period of one and two years, respectively. Analyses with a lag period are often used in pharmacoepidemiologic studies of cancer outcomes to take cancer latency and detection bias into account.^13^ We also conducted a modified intention-to-treat analysis, mainly to account for the possibility that a proportion of patients could have switched from DPP4 inhibitors to GLP1 receptor agonists during follow-up (a potential thyroid cancer risk with GLP1 receptor agonists could then spill over into the group classified as DPP4 inhibitors according to the intention-to-treat definition for drug use, which could lead to an underestimation of risk of thyroid cancer with GLP1 receptor agonists). In this analysis, a further censoring criterion was applied so that patients were censored from follow-up on the date of switch to, or add on of, the other study drug (ie, follow-up among patients treated with DPP4 inhibitors was censored on the date of a GLP1 receptor agonist prescription, and vice versa).

We conducted additional analyses using an “as-treated” definition for drug use, in which patients were censored at treatment discontinuation or switch to the other study drug. Three distinct as-treated analyses were conducted. In these analyses, treatment duration was based on the estimated number of days covered by filled prescriptions, allowing for up to 30 days, one year, and two years between prescriptions (gap period) and after the last prescription (tail period). The tail period allowed for various degrees of latency until the occurrence of the outcome event after treatment discontinuation. We adjusted for potential dependent censoring of patients who changed treatment status during follow-up with inverse probability of censoring weighting.^14^ Follow-up was defined in discrete time intervals of the same length: 90 days. Censoring and outcome events were identified by the follow-up interval in which they occurred. The censoring weight for a certain patient and time interval was calculated as the inverse of the conditional probability of not being censored in the previous interval. Therefore, patients at risk were assigned weights at each time interval so that they also represented the patients who were censored because of treatment changes, including their distribution of risk factors for the outcome. The final weights were calculated as the product of the baseline propensity score weight and all inverse probability of censoring weights leading up to and including the time interval analysed. The final weights were truncated at the 1st and 99th percentile to avoid using extreme weights. The censoring model was estimated with logistic regression and we adjusted for baseline drug use, the time of censoring (including second and third degree polynomials), and all baseline variables from the propensity score model. We estimated odds ratios of the outcome with weighted, pooled logistic regression where all follow-up intervals were included.^15^ In these analyses, the odds ratio for drug use can be interpreted as a hazard ratio because it is estimated over discrete follow-up intervals. The only covariates that were included in the model were drug use and time interval (including second and third degree polynomials).

The propensity score used in the main analysis did not include calendar year; in an additional analysis, we adjusted the hazard ratio for calendar year in three year intervals in a multivariable model (in addition to propensity score weighting). In further analyses, we included calendar year in the propensity score and estimated calendar time specific propensity scores (with the estimation of the scores stratified by calendar time period in four year intervals and country). The cohort for the main analysis excluded patients with any non-thyroid cancer in the previous year before cohort entry; we conducted an additional analysis in which patients with any non-thyroid cancer at any time before cohort entry were excluded. Finally, to assess the impact of accounting for death as a competing event for thyroid cancer, we conducted post hoc robustness analyses. We estimated weighted cumulative incidence functions in which we accounted for any cause death as a competing event,^16^ in contrast to the main analysis in which we estimated Kaplan-Meier functions where patients were censored at death. We estimated risk ratios at five and 10 years, and to enable assessment of the impact of taking death into account as a competing event, we present risk ratios for analyses in which death was a censoring event and a competing event, respectively. A bootstrap with 2000 resamples was used to estimate 95% confidence intervals.

### Patient and public involvement

This study investigated a well defined research question on a potential adverse event of GLP1 receptor agonists that is broadly recognised for its potential impact on patients, and that has been raised as a drug safety concern by drug regulatory agencies. While we support the involvement of patients and the public, there was no funding available for such undertakings in this project and no patients were involved in setting the research question, or in the design, conduct, or interpretation of the study. However, one impetus for the study was clinical encounters in which patients express concerns over drug safety. The study is based on anonymised nationwide register data, and while we will disseminate the results to the public through media, no dissemination of results directly to study participants is planned.

## Results

**Fig 1:**
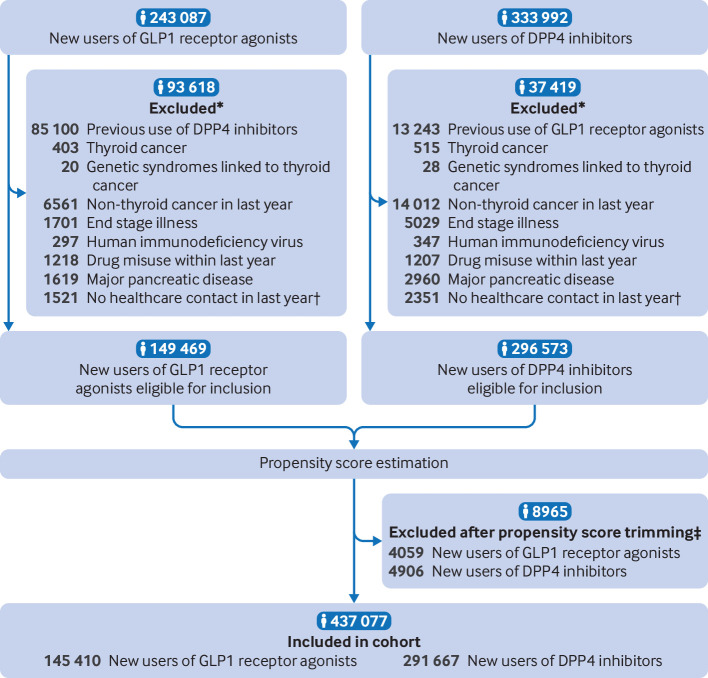
Flowchart of inclusion in study cohort. *Patients could be excluded for more than one reason. †Defined as no specialist care contact or use of any prescription drug. ‡Patients with a propensity score outside the overlapping common distribution of the two groups and the highest 1% and lowest 1% of the common propensity score distribution were excluded (trimming). DPP4=dipeptidyl peptidase 4; GLP1=glucagon-like peptide 1

After exclusions and propensity score trimming, the cohort consisted of 145 410 patients who started GLP1 receptor agonist treatment and 291 667 patients who started DPP4 inhibitor treatment (fig 1). The mean age of the GLP1 receptor agonist group was 57.5 years (standard deviation 12.6 years), and 53.2% were men. The GLP1 receptor agonist and DPP4 inhibitor groups were well balanced on all baseline characteristics after propensity score weighting (fine stratification; table 1).

**Table 1:** Baseline characteristics of study cohort

Characteristics	Before propensity score weighting				After propensity score weighting*		
	GLP1 receptor agonists (n=145 410)	DPP4 inhibitors (n=291 667)	Standardised difference (%)		GLP1 receptor agonists (%)	DPP4 inhibitors (%)	Standardised difference (%)
Country†							
Denmark	62 496 (43.0)	87 504 (30.0)	—		43.0	41.4	—
Norway	10 059 (6.9)	61 780 (21.2)	—		6.9	7.2	—
Sweden	72 855 (50.1)	142 383 (48.8)	—		50.1	51.4	—
Men	77 412 (53.2)	175 033 (60.0)	13.7		53.2	54.0	1.5
Mean age (SD), years‡	57.5 (12.6)	63.5 (11.8)	—		57.5 (12.6)	58.0 (12.6)	—
Age group (years)							
18 to <35	7393 (5.1)	4479 (1.5)	19.9		5.1	4.8	1.3
35 to <40	6290 (4.3)	5415 (1.9)	14.3		4.3	4.1	1.2
40 to <45	10 120 (7.0)	11 234 (3.9)	13.8		7.0	6.8	0.7
45 to <50	14 963 (10.3)	19 277 (6.6)	13.3		10.3	10.0	1.1
50 to <55	19 734 (13.6)	28 710 (9.8)	11.6		13.6	13.4	0.4
55 to <60	21 609 (14.9)	36 576 (12.5)	6.8		14.9	15.0	0.5
60 to <65	21 500 (14.8)	44 067 (15.1)	0.9		14.8	14.8	0.2
65 to <70	19 029 (13.1)	47 204 (16.2)	8.8		13.1	13.3	0.6
70 to <75	14 214 (9.8)	43 555 (14.9)	15.7		9.8	10.1	1.2
75 to <80	7852 (5.4)	32 005 (11.0)	20.4		5.4	5.7	1.4
80 to <85	2706 (1.9)	19 145 (6.6)	23.6		1.9	1.9	0.3
Place of birth							
Scandinavia	123 855 (85.2)	239 840 (82.2)	8.0		85.2	84.9	0.8
Rest of Europe	8623 (5.9)	20 154 (6.9)	4.0		5.9	6.0	0.3
Outside Europe	12 932 (8.9)	31 673 (10.9)	6.6		8.9	9.1	0.7
Civil status							
Living with partner	80 307 (55.2)	164 733 (56.5)	2.5		55.2	54.1	2.3
Not living with partner	64 722 (44.5)	125 618 (43.1)	2.9		44.5	45.6	2.3
Data missing	381 (0.3)	1316 (0.5)	3.2		0.3	0.3	0.4
Medical history							
Cardiovascular disease	33 313 (22.9)	76 604 (26.3)	7.8		22.9	24.2	2.9
Diabetes complications	40 235 (27.7)	80 629 (27.6)	0.1		27.7	29.6	4.4
Obesity diagnosis	23 954 (16.5)	19 264 (6.6)	31.3		16.5	17.3	2.2
Thyroid disorders	6527 (4.5)	10 174 (3.5)	5.1		4.5	4.5	0.0
Thyroid biopsy§	343 (0.2)	471 (0.2)	1.7		0.2	0.2	0.3
Thyroid surgery§	400 (0.3)	513 (0.2)	2.1		0.3	0.3	0.4
Hyperthyroidism treatment in past year§	838 (0.6)	1531 (0.5)	0.7		0.6	0.6	0.3
Thyroid hormone replacement therapy in past year	13 782 (9.5)	24 252 (8.3)	4.1		9.5	9.6	0.3
Diabetes drugs in past six months							
Metformin	98 157 (67.5)	232 031 (79.6)	27.6		67.5	69.4	4.1
Insulin	44 150 (30.4)	36 801 (12.6)	44.2		30.4	33.0	5.6
SGLT2 inhibitors	15 766 (10.8)	11 569 (4.0)	26.5		10.8	12.3	4.5
Sulphonylureas	18 331 (12.6)	68 697 (23.6)	28.7		12.6	13.8	3.6
Other antidiabetics	4246 (2.9)	15 054 (5.2)	11.4		2.9	3.1	1.0
No of prescription drugs in past year							
<5	40 050 (27.5)	85 979 (29.5)	4.3		27.5	25.4	4.8
6-10	55 823 (38.4)	118 396 (40.6)	4.5		38.4	38.6	0.4
11-15	31 059 (21.4)	57 139 (19.6)	4.4		21.4	22.2	2.1
≥16	18 478 (12.7)	30 153 (10.3)	7.4		12.7	13.8	3.2
No of outpatient physician visits in past year							
0	48 596 (33.4)	117 418 (40.3)	14.2		33.4	32.7	1.5
1-3	64 008 (44.0)	124 245 (42.6)	2.9		44.0	44.2	0.3
≥4	32 806 (22.6)	50 004 (17.1)	13.6		22.6	23.1	1.3
No of hospital admissions in past year							
0	113 718 (78.2)	232 437 (79.7)	3.6		78.2	77.3	2.1
1-2	25 396 (17.5)	46 952 (16.1)	3.7		17.5	18.0	1.4
≥3	6296 (4.3)	12 278 (4.2)	0.6		4.3	4.7	1.6

Data are numbers (%) unless stated otherwise.

DPP4=dipeptidyl peptidase 4; GLP1=glucagon-like peptide 1; SD=standard deviation; SGLT2=sodium-glucose cotransporter 2.

* Propensity score fine stratification weighting.

† Country shown descriptively; propensity score was estimated stratified by country.

‡ Mean age shown descriptively; age group by categories was included in propensity score.

§ Variable not available in Norwegian data; percentages shown are calculated on Swedish and Danish data.

The most common individual GLP1 receptor agonist was liraglutide (57.3%), followed by semaglutide (32.9%), dulaglutide (4.9%), exenatide (4.1%), and lixisenatide (0.9%). The mean follow-up time among patients who started GLP1 receptor agonists was 3.9 (standard deviation 3.5) years; 25% of patients were followed for 6.1 years or longer. The mean follow-up time varied by specific drug: liraglutide 5.2 (3.4) years, semaglutide 1.1 (0.8) years, dulaglutide 2.9 (1.8) years, exenatide 9.1 (3.9) years, and lixisenatide 4.0 (1.7) years. The mean follow-up time among patients who started DPP4 inhibitors was 5.4 (3.5) years. Assessing the proportional hazards assumption, there was no significant interaction between follow-up time and use of GLP1 receptor agonists (P=0.82).

**Fig 2:**
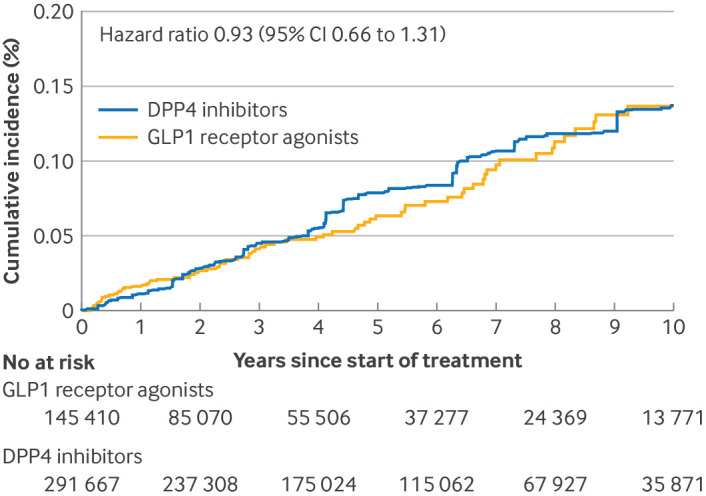
Cumulative incidence of thyroid cancer. The cumulative incidence curve and hazard ratio were adjusted for all variables shown in table 1 using propensity score fine stratification weighting. The cumulative incidence curve was truncated at 10 years because of decreasing numbers of participants and outcome events; maximum follow-up was 14.6 years. CI=95% confidence interval; DPP4=dipeptidyl peptidase 4; GLP1=glucagon-like peptide 1

Figure 2 shows the cumulative incidence of thyroid cancer among the GLP1 receptor agonist and DPP4 inhibitor groups. During follow-up, 76 patients with incident thyroid cancer were identified in the GLP1 receptor agonist group and 184 in the DPP4 inhibitor group; the incidence rates were 1.33 and 1.46 per 10 000 person years, respectively. The hazard ratio was 0.93 (95% confidence interval 0.66 to 1.31; table 2).

**Table 2:** GLP1 receptor agonist use and risk of thyroid cancer

Analysis	GLP1 receptor agonists (n=145 410)			DPP4 inhibitors (n=291 667)		Hazard ratio (95% CI)*	Rate difference (95% CI) per 10 000 person years*
	No of events	Rate per 10 000 person years*		No of events	Rate per 10 000 person years*		
Main analysis (any thyroid cancer)	76	1.33		184	1.46	0.93 (0.66 to 1.31)	−0.13 (−0.61 to 0.36)
Subtypes of thyroid cancer							
Papillary	53	0.93		114	1.04	0.92 (0.61 to 1.39)	−0.11 (−0.53 to 0.31)
Follicular	16	0.28		47	0.27	0.99 (0.47 to 2.08)	0.01 (−0.19 to 0.21)
Medullary	4	0.07		11	0.07	1.19 (0.37 to 3.86)	0.00 (−0.09 to 0.09)
Other	5	0.09		15	0.05	1.51 (0.44 to 5.20)	0.04 (−0.05 to 0.12)

Total crude number of person years in main analysis was 569 538 in the GLP1 receptor agonist group and 1 580 409 in the DPP4 inhibitor group.

CI=confidence interval; DPP4=dipeptidyl peptidase 4; GLP1=glucagon-like peptide 1.

* Rates, rate differences, and hazard ratios adjusted for all variables shown in table 1 using propensity score fine stratification weighting.

Papillary thyroid cancer was the most common thyroid cancer subtype, followed by follicular, medullary, and other types. No significant increases in risk of any of the thyroid cancer subtypes were identified with GLP1 receptor agonist use, although the number of events was relatively small and the estimates for subtypes other than papillary were imprecise (table 2).

Table 3 shows additional analyses of GLP1 receptor agonist use and risk of thyroid cancer. For the analyses with different lag periods after starting treatment, the hazard ratio for thyroid cancer was 0.83 (95% confidence interval 0.56 to 1.22) with a one year lag and 0.90 (0.58 to 1.38) with a two year lag. The hazard ratio for the first year after starting treatment was 1.47 (0.74 to 2.93). Similar to the main analysis, no significant associations between GLP1 receptor agonists versus DPP4 inhibitors and risk of thyroid cancer were observed in additional analyses which used an alternative modified intention-to-treat definition for drug use; included additional adjustment for calendar year; included calendar year in the propensity score; applied calendar time specific propensity scores; and excluded patients with any previous cancer at any time before cohort entry.

**Table 3:** Additional analyses of GLP1 receptor agonist use and risk of thyroid cancer

Additional analyses	No of patients	No of events	Rate per 10 000 person years*	Hazard ratio (95% CI)*
**Time periods**				
1 year lag†: GLP1 receptor agonists	108 284	55	1.24	0.83 (0.56 to 1.22)
1 year lag†: DPP4 inhibitors	265 541	150	1.53	Reference
2 year lag†: GLP1 receptor agonists	85 070	45	1.29	0.90 (0.58 to 1.38)
2 year lag†: DPP4 inhibitors	237 308	117	1.48	Reference
Risk during first year‡: GLP1 receptor agonists	145 410	21	1.67	1.47 (0.74 to 2.93)
Risk during first year‡: DPP4 inhibitors	291 667	34	1.13	Reference
**Modified intention-to-treat analysis§**				
GLP1 receptor agonists	145 410	68	1.32	0.99 (0.68 to 1.44)
DPP4 inhibitors	291 667	147	1.35	Reference
**As-treated analysis with 90 day gap and tail¶**				
All follow-up: GLP1 receptor agonists	145 410	38	1.44	1.37 (0.84 to 2.23)
All follow-up: DPP4 inhibitors	291 667	76	1.11	Reference
1 year lag†: GLP1 receptor agonists	72 430	17	1.10	0.99 (0.50 to 1.97)
1 year lag†: DPP4 inhibitors	187 617	49	1.12	Reference
Risk during first year‡: GLP1 receptor agonists	145 410	21	1.94	2.19 (0.98 to 4.85)
Risk during first year‡: DPP4 inhibitors	291 667	27	1.10	Reference
**As-treated analysis with 1 year gap and tail¶**				
All follow-up: GLP1 receptor agonists	145 410	52	1.36	1.04 (0.69 to 1.57)
All follow-up: DPP4 inhibitors	291 667	117	1.41	Reference
1 year lag†: GLP1 receptor agonists	105 254	31	1.20	0.78 (0.46 to 1.33)
1 year lag†: DPP4 inhibitors	249 520	86	1.55	Reference
Risk during first year‡: GLP1 receptor agonists	145 410	21	1.69	1.94 (0.92 to 4.08)
Risk during first year‡: DPP4 inhibitors	291 667	31	1.11	Reference
**As-treated analysis with 2 year gap and tail¶**				
All follow-up: GLP1 receptor agonists	145 410	56	1.31	0.99 (0.67 to 1.46)
All follow-up: DPP4 inhibitors	291 667	127	1.38	Reference
1 year lag†: GLP1 receptor agonists	105 254	35	1.15	0.76 (0.46 to 1.23)
1 year lag†: DPP4 inhibitors	249 520	96	1.49	Reference
Risk during first year‡: GLP1 receptor agonists	145 410	21	1.69	1.94 (0.92 to 4.09)
Risk during first year‡: DPP4 inhibitors	291 667	31	1.11	Reference
Additional adjustment for calendar year**				
GLP1 receptor agonists	145 410	76	1.33	0.93 (0.66 to 1.31)
DPP4 inhibitors	291 667	184	1.46	Reference
**Calendar year included in propensity score**				
GLP1 receptor agonists	145 111	80	1.38	0.81 (0.53 to 1.24)
DPP4 inhibitors	291 925	184	1.73	Reference
**Calendar time specific propensity score**				
GLP1 receptor agonists	145 091	81	1.40	0.80 (0.50 to 1.27)
DPP4 inhibitors	291 413	182	1.79	Reference
**Patients with any previous cancer excluded**				
GLP1 receptor agonists	136 601	70	1.29	0.93 (0.65 to 1.32)
DPP4 inhibitors	269 600	165	1.42	Reference
**SGLT2 inhibitors as comparator group**				
GLP1 receptor agonists	111 744	40	1.21	1.16 (0.65 to 2.05)
SGLT2 inhibitors	148 179	26	1.07	Reference

CI=confidence interval; DPP4=dipeptidyl peptidase 4; GLP1=glucagon-like peptide 1; SGLT2=sodium-glucose cotransporter 2.

* Rates and hazard ratios adjusted for all variables shown in table 1 using propensity score fine stratification weighting.

† Analysis in which follow-up is lagged by 1 and 2 years, respectively, and all follow-up beyond the first and second year, respectively, is analysed.

‡ Analysis restricted to follow-up time during first year since starting drug; that is, risk during time period that is lagged in an analysis with 1 year lag period.

§ Patients treated with GLP1 receptor agonists were censored on starting DPP4 inhibitors, and vice versa.

¶ Analysis censored at treatment discontinuation or switch to other study drug, accounting for censoring through inverse probability of censoring weighting.

** Multivariable adjustment for calendar year in 3 year intervals, in addition to propensity score weighting.

In additional analyses that used an alternative as-treated definition for drug use, the mean follow-up time among patients who started GLP1 receptor agonists was 1.8 years in the analysis with 90 day gap and tail periods, 2.6 years in the analysis with one year gap and tail periods, and 2.9 years in the analysis with two year gap and tail periods. The hazard ratios for the full follow-up period in these analyses was 1.37 (95% confidence interval 0.84 to 2.23), 1.04 (0.69 to 1.57), and 0.99 (0.67 to 1.46), respectively. The pattern of association reflected that of the main intention-to-treat analysis, with point estimates less than 1.0 in the as-treated analyses with a one year lag and with increased hazard ratios in the as-treated analyses restricted to the first year after starting treatment. In post hoc robustness analyses assessing the impact of competing risk of death, the risk ratios for thyroid cancer at five years of follow-up were 0.80 (0.58 to 1.14) with death as a censoring event and 0.82 (0.59 to 1.16) with death as a competing event. At 10 years of follow-up, the risk ratios for thyroid cancer were 1.00 (0.72 to 1.37) with death as a censoring event and 1.01 (0.74 to 1.39) with death as a competing event.

For the additional analysis that compared GLP1 receptor agonists with SGLT2 inhibitors, the flowchart for inclusion and the baseline characteristics are shown in efigure 1 and etable 4, respectively. This analysis included a cohort of 111 744 patients who started GLP1 receptor agonist treatment and 148 179 patients who started SGLT2 inhibitor treatment. GLP1 receptor agonist use was not associated with increased risk of thyroid cancer compared with SGLT2 inhibitor use (hazard ratio 1.16, 95% confidence interval 0.65 to 2.05; table 3, efigure 2).

## Discussion

### Principal findings

In this cohort study using nationwide data from three countries, GLP1 receptor agonist use was not associated with a substantially increased risk of thyroid cancer over a mean follow-up of 3.9 years. The study was based on more than 145 000 patients who started GLP1 receptor agonist treatment and had high statistical precision; given the upper limit of the confidence interval, the findings are incompatible with an increased relative risk of thyroid cancer of more than 31%. In absolute terms, this translates to no more than 0.36 excess events per 10 000 person years, which should be interpreted against the background incidence of 1.46 per 10 000 person years in the comparator group in the study population. Findings were neutral, but less precise for specific subtypes of thyroid cancer, including medullary thyroid cancer, and robust in several additional analyses, including when an alternative comparator group was used. However, the study cannot exclude a small increase in risk.

### Comparison with other studies

Although pharmacovigilance studies have found increased reporting rates for thyroid cancer with GLP1 receptor agonists,^2^ ^3^ disproportionality analyses are designed to detect potential safety signals but are not intended to make causal conclusions. Given that thyroid cancer is mentioned in the product label as a potential adverse event, spontaneous reporting might have been driven by physician and public awareness. While meta-analyses of randomised trials have generated point estimates >1, the confidence intervals have been wide owing to limited sample size and short follow-up time, which limits the assessment of rare cancer events.^4^ ^5^ The largest meta-analysis was based on 43 124 patients exposed to GLP1 receptor agonists in trials with a median duration of only 75 weeks, and the confidence interval was consistent with up to twofold increase in risk. These meta-analyses represent post hoc analyses of secondary safety events and individual trials have not included thyroid cancer as a primary outcome.

A recent nested case-control study based on a French national health insurance system database reported a significant association between thyroid cancer and GLP1 receptor agonist use (hazard ratio 1.46, 95% confidence interval 1.23 to 1.74).^7^ However, the comparator in that study was non-use of GLP1 receptor agonists, rather than an active comparator; exposure groups might have been misaligned on important baseline characteristics, potentially leading to confounding. Point estimates in that study were of similar magnitude for duration of drug use <1 year, 1-3 years, and >3 years.^7^ Given that a potential effect of GLP1 receptor agonists on thyroid cancer is unlikely to emerge after short term use, these findings might indicate the presence of confounding. An alternative explanation for the increased risk observed in the French study, emerging early and staying at similar magnitude with increasing duration of use, could be detection bias.^17^ Our additional analysis indicated a nominally increased risk restricted to the first year after starting treatment, which might be consistent with an increased detection of thyroid cancer among patients using GLP1 receptor agonists. In contrast to the French study, a recent study based on nationwide data from Korea used an active-comparator new user cohort design and propensity score weighting to control for confounding. Applying a one year lag period, the hazard ratio for thyroid cancer comparing GLP1 receptor agonists with SGLT2 inhibitors was 0.98 (95% confidence interval 0.62 to 1.53).^18^

### Strengths and limitations

This study was based on a large unselected study population from routine clinical practice, which was derived from three Scandinavian countries that have universal access to tax funded healthcare, and high quality data with nationwide coverage, including dedicated cancer registers. Furthermore, we used robust pharmacoepidemiologic methods. These strengths substantiate the generalisability of results, reduce concerns about selection and information biases, and support the internal validity of the study. Therefore, this study adds to the available evidence about GLP1 receptor agonist use and risk of thyroid cancer, and supports the conclusion of a recent European Medicines Agency investigation that the available evidence does not support a causal association between GLP1 receptor agonist use and thyroid cancer.^9^

In our study, drug use in the main analysis was defined according to the observational analogue of intention to treat (ie, from the first filled prescription onwards) to provide the least conservative definition. Because cancer takes time to develop and manifest clinically, and because the nature and timing of a potential association between GLP1 receptor agonists and thyroid cancer are not known, the definition allowed identification of thyroid cancer events while on treatment and after treatment had stopped. Additional analyses were conducted with a modified intention-to-treat definition for drug use so that patients were censored from follow-up on the date of switch to, or add on of, the other study drug (ie, follow-up among patients treated with DPP4 inhibitors was censored on the date of a GLP1 receptor agonist prescription, and vice versa). This approach mainly accounted for the possibility that a proportion of patients could have switched from DPP4 inhibitors to GLP1 receptor agonists during follow-up (a potential thyroid cancer risk with GLP1 receptor agonists could spill over into the group classified as receiving DPP4 inhibitors according to the intention-to-treat definition for drug use, which might lead to an underestimation of thyroid cancer risk with GLP1 receptor agonists). However, the estimate of the modified intention-to-treat analysis was not substantially different from that of the main analysis. We conducted additional analyses with an as-treated definition for drug use, which assessed the risk of thyroid cancer while on treatment with GLP1 receptor agonists. Similar to the pattern observed in the main intention-to-treat analysis, a nominally increased risk restricted to the first year of treatment was observed in the as-treated analyses, whereas the point estimates were <1.0 in as-treated analyses with a one year lag. The findings of the as-treated analysis are consistent with an increased detection of thyroid cancer in the immediate period after starting GLP1 receptor agonist treatment, or other bias, given that a risk increase restricted to the first year of treatment is unlikely to be biologically plausible.

The study has limitations. Although the mean follow-up among GLP1 receptor agonist users was 3.9 years, because of cancer latency,^13^ extended follow-up might be needed to detect an increased risk. However, 25% of the study participants were followed for 6.1 years or longer and the cumulative incidence curves showed no signs of an emerging risk increase with up to 10 years of follow-up. We investigated GLP1 receptor agonists at drug class level and liraglutide and semaglutide were the most commonly used individual drugs in the study. An avenue for future work is to assess thyroid cancer risk by individual GLP1 receptor agonist. Although we analysed thyroid cancer subtypes and observed neutral results for each of the subtypes including medullary thyroid cancer, the number of events in this analysis was relatively small and the estimates for subtypes other than papillary were imprecise. Similarly, we could not assess smaller subgroups of patients, including those with previous cancers or conditions associated with increased risk of thyroid cancer.

Potential limitations with real world studies of drug effects include the possibility of confounding and time related biases. To minimise these risks, we used an active-comparator new user study design.^11^ This design aligns patients at a uniform point in time to start follow-up (starting drug treatment), ensures correct temporality for covariate and drug use assessment, and balances patients on baseline characteristics by selecting a comparator drug that is used for the same indication and at similar stages of disease. We chose DPP4 inhibitors as the primary active comparator. These drugs were introduced at around the same time as the GLP1 receptor agonists and are similarly used as second and third line drugs for diabetes; therefore, they probably represent the most suitable active comparator over the study period overall. We also conducted an additional analysis with SGLT2 inhibitors as the comparator. Similar to GLP1 receptor agonists, recent guidelines recommend these drugs for cardiovascular risk reduction among patients with diabetes, and the use of both drug classes has increased in recent years.^19^ ^20^

Finally, we controlled for a number of baseline characteristics, including sociodemographic characteristics, medical history, other diabetes treatment, and markers of healthcare use, through propensity score weighting. Despite these measures, unmeasured or residual confounding cannot be ruled out given the observational design of this study. Of specific concern would be factors that obscure a true increased risk of thyroid cancer with GLP1 receptor agonists, thereby influencing the interpretation of the study. Such factors would have to be protective against thyroid cancer and be more prevalent in the GLP1 receptor agonist group than in the comparator groups, or increase the risk of thyroid cancer and be less prevalent in the GLP1 receptor agonist group than in the comparator groups. The only known factor that is potentially associated with a reduced risk of thyroid cancer is smoking; however, in our previous works using nationwide Scandinavian data, the prevalence of current smoking has been similar among patients treated with GLP1 receptor agonists and those treated with DPP4 inhibitors and SGLT2 inhibitors, respectively.^21^ ^22^ Factors that are associated with increased risk of thyroid cancer include female sex, age, ethnicity, obesity, taller height, family history of thyroid cancer, ionising radiation during childhood, iodine intake, and potentially diabetes duration.^23^ ^24^ ^25^ ^26^ While information on family history, ionising radiation, iodine intake and height were not available for this study, we adjusted for sex, age, place of birth, and obesity diagnosis. Before propensity score weighting, the proportion of patients with an obesity diagnosis was more than double in the GLP1 receptor agonist group than in both comparator groups. Although body mass index data were not available for this cohort, our findings suggest that any unmeasured obesity is likely to have been more prevalent in the GLP1 receptor agonist group, with any associated bias potentially moving the estimate towards increased risk of thyroid cancer.

### Conclusions

In conclusion, this large cohort study found that GLP1 receptor agonist treatment was not associated with a substantially increased risk of thyroid cancer over a mean follow-up of 3.9 years. In the main analysis, which compared GLP1 receptor agonists with DPP4 inhibitors, the upper limit of the confidence interval was consistent with no more than a 31% increase in relative risk.

**What is already known on this topic**

During development of glucagon-like peptide 1 (GLP1) receptor agonists, studies in rodents showed increased rates of thyroid tumours
Subsequent reports based on pharmacovigilance data, post hoc analyses of randomised trials, and findings from observational studies indicate a potential link between GLP1 receptor agonists and thyroid cancer, although not conclusively

**What this study adds**

This cohort study used nationwide register data from Sweden, Denmark, and Norway to investigate the risk of thyroid cancer among patients treated with GLP1 receptor agonists
Over a mean follow-up of 3.9 years, GLP1 receptor agonist treatment was not associated with a substantially increased risk of thyroid cancer compared with dipeptidyl peptidase 4 (DPP4) inhibitors, and in an additional analysis, sodium-glucose cotransporter 2 inhibitors
Although small risk increases cannot be excluded, in the main analysis comparing GLP1 receptor agonists with DPP4 inhibitors, the upper limit of the confidence interval was consistent with no more than a 31% increase in relative risk

## Data Availability

No additional data available. The data analysed in this study were based on Scandinavian nationwide register. Individual level data from the registers can only be accessed through secure servers and only export of aggregated data, as presented in research articles, is allowed. Permission to access data can be made only after fulfilling specific requirements to safeguard the anonymity of the study participants and other data safety issues. For these reasons, data cannot be made generally available.

## Web extra

Extra material supplied by authors

Web appendix: Supplementary appendix

## Notes

Contributors: BP, VW, and PU initiated the study. BP and VW had full access to all the data in the study and take responsibility for the integrity of the data and the accuracy of the data analysis. BP drafted the manuscript. VW performed the statistical analysis. All authors contributed to the design of the study; the acquisition, analysis, and interpretation of data; and to the critical revision of the manuscript for important intellectual content. BP supervised the study and is the guarantor. The corresponding author attests that all listed authors meet authorship criteria and that no others meeting the criteria have been omitted.

Transparency: The lead author (the manuscript’s guarantor) affirms that the manuscript is an honest, accurate, and transparent account of the study being reported; that no important aspects of the study have been omitted; and that any discrepancies from the study as planned (and, if relevant, registered) have been explained.

Dissemination to participants and related patient and public communities: The study is based on anonymised nationwide register data, and while the authors will disseminate the results to the public through media, no dissemination of results directly to study participants is planned. The results of this research will be disseminated to the public via the websites and social media of the authors’ institutes, authors’ social media, and via a press release; presented to the scientific community at a pharmacoepidemiology conference; and shared with clinicians caring for patients with diabetes.

Provenance and peer review: Not commissioned; externally peer reviewed.
