# Supplementary material for: Glucagon-like peptide 1 receptor agonist use and risk of thyroid cancer: Scandinavian cohort study
Source: BMJ. 2024 Apr 10;385:e078225. doi: 10.1136/bmj-2023-078225 (PMC11004669; doi:10.1136/bmj-2023-078225)
Supplement: Supplementary file 1 — Web appendix: Supplementary appendix [file pasb078225.ww.pdf]

SUPPLEMENTARY APPENDIX

Glucagon-like peptide 1 receptor agonist use and risk of thyroid cancer: Scandinavian cohort study

Pasternak et al.

eTable 1. Exclusions Criteria with Definitions

**Criterion**	**Codes (ICD-10, NCSP, or ATC))**<br><br>**Inpatient/outpatient settings, any position**
Thyroid cancer at any time before cohort entry	ICD-10: C73 (from cancer and patient registers)
Any cancer record (except non-melanoma skin cancer) in last year before cohort entry	ICD-10: C00-C43, C45-C97, D45 (from cancer and patient registers)
Endstage illness (severe malnutrition, cachexia, dementia, coma, end-stage renal disease, organ transplant) at any time before cohort entry[a]	ICD-10: E40-E43, F00-F03, G30, R40.2, R64, N18.5, N18.6, Z99.2, Z49, T86, Y83.0, Z48.2, Z94<br><br>NCSP: KAS, FQA, FQB, JJC, GDG; Sweden: DR012, DR013, DR014, DR015, DR016, DR023, DR024, DR055, DR056, DR060, DR061; Denmark: (B)JFD, (B)JFZ; Norway: A0093, A0094, JAGD30, JAGD31, JAGD32, JAGD50, JAK10, PHGX00, PHGX05, RXGD05, RXGD20, RXGD25, TJA33<br><br>ATC: N06D
HIV at any time before cohort entry[a]	ICD-10: B20, B21-B24, R75, Z21, O98.7
Drug misuse, alcohol abuse and nicotine dependence in last year before cohort entry	ICD-10: F10-F19, R78.1-R78.5, T40[b]; ATC: N07BB, N07BC
Major pancreatic disease (chronic pancreatitis [defined by pancreatic enzyme substitution prescription within last year or diagnosis at any time before cohort entry], pancreatic cancer, major pancreatic surgery at any time before cohort entry)[a]	ICD-10: C25, K86.0, K86.1, K90.3, Z94.83; NCSP: JLC, JLE; ATC: A09AA02
Genetic syndromes associated with thyroid cancer at any time before cohort entry[a]	ICD-10: Q78.1, Q85.8, Q85.9
No health care contact in last year before cohort entry	Neither outpatient specialist care contact, hospitalization, nor use of any prescription drug

Abbreviations: ICD, International Classification of Diseases; NCSP, NOMESCO Classification of Surgical Procedures; ATC, Anatomical Therapeutic Chemical

a. 10 years look-back in Sweden, Denmark; 2 years look-back in Norway.
b. Not available in Norway.

eTable 2. Subtypes of Thyroid Cancer as Defined by ICD-O/3 Morphology Codes in the Cancer Registers

Subtype	ICD-O/3 morphology code
Follicular	83301, 83303, 83313, 83353, 83403, 82903, 83463*
Medullary	83453, 85103, 83463*
Papillary	80503, 82603, 83413, 83443
Other	80103, 80213, 81403, 814039, 85893, 80313, 84803, 84303, 80203, 80703, 83503, 80413, 80123, 82303, 824633, 83363, 83373, 959133, 96803

*code 83463 defines mixed medullary-follicular thyroid carcinoma and was therefore included in both follicular and medullary thyroid cancer categories

eTable 3. Covariate Definitions

Covariate	Definitions
Age	5-year intervals
Sex	Women/men
Interaction between age and sex	Age category x sex
Place of birth	Scandinavia; Rest of Europe; Outside Europe
Civil status	Living with partner; Not living with partner; Missing
Medical history (5 yr look-back in Sweden and Denmark; 2 year look-back in Norway)	
Cardiovascular disease	ICD-10: I09.8, Z95.1, Z95.5, I11, I20, I21, I22, I24, I25, I130, I132, I34-I37, I42, I43, I44-I47, I48, I49, I50, I60-I69, I70, I72, I73, I74, I77, J81, G45 (excl G453, G454), G46, K550, K551, E115, E145, E135; Procedure codes: FNA, FNB, FNC, FND, FNE, FNG, FNP02, FNP12, FNQ05, FNQ12, FNR22, PDA-PDW, PFA-PFW, PEA-PEW
Diabetes complications	ICD-10: E110, E111, E112, E113, E114, E116, E117, E118, E130, E131, E133, E134, E136, E137, E138, E140, E141, E142, E143, E144, E146, E147, E148, E160, E161, E162, G990, G590, G632, H280[a], H358, H360, I120, I131, I132, L984, M142, M146, M908, N00-08, N10-N16, N17, N18, N19, N20-N23, N25-N29; Procedure codes[a]: CKC10, CKC12, CKC15, CKD65
Obesity diagnosis	ICD-10: E66
Thyroid disorders	ICD-10: E00-E07
Thyroid biopsy[a]	Procedure codes: BAA00, TBA00, TBA10, TBA20
Thyroid surgery[a]	Procedure codes: BAA (not BAA00)
Hyperthyroidism treatment in last yr.[a]	ATC: H03BA02, H03BB02
Thyroid hormone replacement therapy in last yr.	ATC: H03AA01, H03AA02
Diabetes drugs in last 6 mo.	
Metformin	ATC: A10BA02, A10BD02, A10BD03, A10BD05, A10BD07, A10BD08, A10BD10, A10BD11, A10BD13, A10BD14, A10BD15, A10BD16, A10BD20, A10BD22, A10BD23, A10BD25
Insulin	ATC: A10A
SGLT2 inhibitors	ATC: A10BK01, A10BK02, A10BK03, A10BK04, A10BD15, A10BD16, A10BD19, A10BD20, A10BD21, A10BD23, A10BD24, A10BD25
Sulphonylureas	ATC: A10BB, A10BD01, A10BD02, A10BD04, A10BD06
Other antidiabetics (glitazones, glinides, acarbose)	ATC: A10BG, A10BX, A10BF01, A10BF02, A10BD03, A10BD04, A10BD05, A10BD06, A10BD09, A10BD14
Health care utilization in last yr.	

Number of distinct prescription drugs	<5, 6-10, 11-15, ≥16
Number of outpatient physician visits	0, 1-3, ≥4
Number of hospital admissions	0, 1-2, ≥3

[a] covariate/code not available in Norwegian data

eTable 4. Baseline Characteristics of Study Cohort for Additional Analysis Comparing GLP1 receptor agonists vs. SGLT2 Inhibitors

	Before propensity score weighting			After propensity score weighting*		
	**GLP1 receptor agonists (n=111,744)**	**SGLT2 inhibitors (n=148,179)**	**Standard-ized difference (%)**	**GLP1 receptor agonists**	**SGLT2 inhibitors**	**Standard-ized difference (%)**
	n (%)	*n (%)*		*%*	*%*	
Country[#]						
Denmark	37,828 (33.9)	46,134 (31.1)	-	33.9	33.3	-
Norway	13,143 (11.8)	26,121 (17.6)	-	11.8	11.8	-
Sweden	60,773 (54.4)	75,924 (51.2)	-	54.4	54.9	-
Men	61,796 (55.3)	95,111 (64.2)	18.2	55.3	56.0	1.4
Mean (SD) age, yrs.[†]	59.1 (12.5)	62.5 (11.3)	-	59.1 (12.5)	59.3 (12.4)	-
Age group, years						
18 to <35	4451 (4.0)	2212 (1.5)	15.3	4.0	3.7	1.4
35 to <40	3944 (3.5)	2758 (1.9)	10.3	3.5	3.4	0.8
40 to <45	6813 (6.1)	5546 (3.7)	10.9	6.1	5.8	1.0
45 to <50	10,476 (9.4)	10,311 (7.0)	8.8	9.4	9.3	0.1
50 to <55	14,401 (12.9)	16,368 (11.0)	5.7	12.9	13.0	0.2
55 to <60	15,935 (14.3)	20,809 (14.0)	0.6	14.3	14.4	0.5
60 to <65	16,332 (14.6)	23,881 (16.1)	4.2	14.6	14.9	0.8
65 to <70	15,684 (14.0)	24,678 (16.7)	7.3	14.0	14.2	0.4
70 to <75	13,250 (11.9)	22,455 (15.2)	9.7	11.9	11.8	0.1
75 to <80	7572 (6.8)	13,466 (9.1)	8.6	6.8	6.8	0.0
80 to <85	2886 (2.6)	5695 (3.8)	7.2	2.6	2.6	0.3
Place of birth						
Scandinavia	94,559 (84.6)	119,087 (80.4)	11.2	84.6	84.6	0.1
Rest of Europe	6865 (6.1)	11,381 (7.7)	6.1	6.1	6.2	0.0
Outside Europe	10,320 (9.2)	17,711 (12.0)	8.8	9.2	9.3	0.1
Civil status						
Living with partner	60,569 (54.2)	83,813 (56.6)	4.7	54.2	53.6	1.2
Not living with partner	50,882 (45.5)	63,923 (43.1)	4.8	45.5	46.1	1.1
Missing	293 (0.3)	443 (0.3)	0.7	0.3	0.3	0.2
**Medical history**						
Cardiovascular disease	27,198 (24.3)	43,150 (29.1)	10.8	24.3	25.0	1.5
Diabetes complications	34,432 (30.8)	40,989 (27.7)	6.9	30.8	31.8	2
Obesity diagnosis	16,675 (14.9)	10,719 (7.2)	24.7	14.9	15.1	0.4
Thyroid disorders	5077 (4.5)	4917 (3.3)	6.3	4.5	4.5	0.0
Thyroid biopsy[‡]	278 (0.2)	258 (0.2)	1.6	0.2	0.2	0.1
Thyroid surgery[‡]	300 (0.3)	262 (0.2)	1.9	0.3	0.3	0

Hyperthyroidism treatment in last yr. ‡	619 (0.6)	743 (0.5)	0.7	0.6	0.5	0.2
Thyroid hormone replacement therapy in last yr.	10,886 (9.7)	11,412 (7.7)	7.2	9.7	9.8	0.1
**Diabetes drugs in last 6 mo.**						
Metformin	78,155 (69.9)	121,250 (81.8)	28.1	69.9	70.8	1.9
Insulin	38,187 (34.2)	26,999 (18.2)	36.9	34.2	36.0	3.7
DPP4 inhibitors	8379 (7.5)	18,952 (12.8)	17.6	7.5	7.6	0.4
Sulphonylureas	16,957 (15.2)	26,654 (18.0)	7.6	15.2	15.8	1.7
Other antidiabetics	3218 (2.9)	4565 (3.1)	1.2	2.9	2.9	0.3
**Health care utilization in last yr.**						
Prescription drugs, no.						
<5	27,198 (24.3)	40,018 (27.0)	6.1	24.3	23.1	2.9
6-10	43,858 (39.2)	63,542 (42.9)	7.4	39.2	39.4	0.3
11-15	25,449 (22.8)	30,390 (20.5)	5.5	22.8	23.3	1.2
≥16	15,239 (13.6)	14,229 (9.6)	12.6	13.6	14.2	1.7
Outpatient physician visits, no.						
0	36,960 (33.1)	57,408 (38.7)	11.8	33.1	32.9	0.5
1-3	49,252 (44.1)	63,405 (42.8)	2.6	44.1	44.1	0.0
≥4	25,532 (22.8)	27,366 (18.5)	10.8	22.8	23.1	0.6
Hospital admissions, no.						
0	88,645 (79.3)	119,139 (80.4)	2.7	79.3	79.0	0.7
1-2	18,611 (16.7)	23,130 (15.6)	2.8	16.7	16.7	0.3
≥3	4488 (4.0)	5910 (4.0)	0.1	4.0	4.2	1.0

* Propensity score weighting with the fine stratification method

Country shown descriptively; ; the propensity score was estimated stratified by country

† Mean age shown descriptively; age group by categories was included in the propensity score.

‡ Variable not available in Norwegian data; percentages shown are calculated on Swedish and Danish data.

eFigure 1. Flow Chart of Cohort Inclusion – Analysis of GLP1 Receptor Agonists vs. SGLT2 Inhibitors

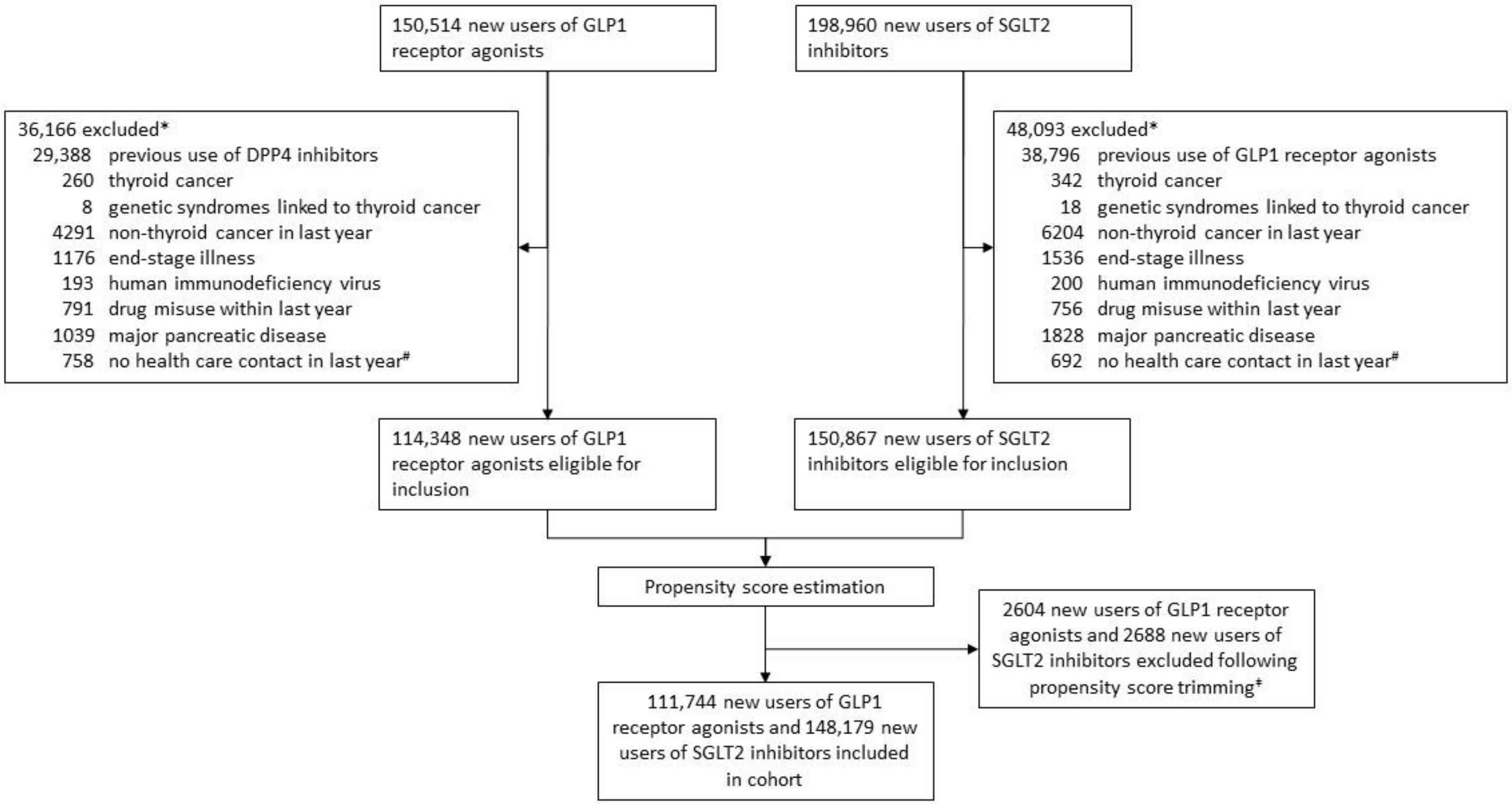

*Patients could be excluded due to more than one reason.
# Defined as neither specialist care contact nor use of any prescription drug.
‡ Trimming of 1% of the study cohort on each end of the common propensity score distribution.

eFigure 2. Cumulative Incidence of Thyroid Cancer in Analysis of GLP1 Receptor Agonists vs. SGLT2 Inhibitors

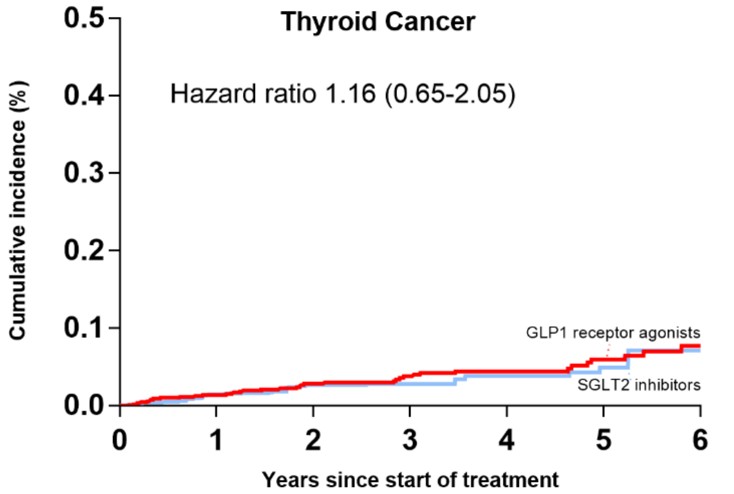

Number at risk

GLP1 receptor agonists	111,744	65,017	35,037	12,435
SGLT2 inhibitors	148,179	73,793	24,588	4607

The cumulative incidence curve and the hazard ratio are adjusted for all variables shown in Table 1 using propensity score fine stratification weighting.
The cumulative incidence curve was truncated at 6 years due to decreasing numbers of participants and outcome events; maximum follow-up in the analysis was 8 years.
